# Sex is determined by XX/XY sex chromosomes in Australasian side-necked turtles (Testudines: Chelidae)

**DOI:** 10.1038/s41598-020-61116-w

**Published:** 2020-03-09

**Authors:** Sofia Mazzoleni, Barbora Augstenová, Lorenzo Clemente, Markus Auer, Uwe Fritz, Peter Praschag, Tomáš Protiva, Petr Velenský, Lukáš Kratochvíl, Michail Rovatsos

**Affiliations:** 1 Department of Ecology, Faculty of Science, Charles University, Viničná 7, Prague, Czech Republic; 2 Museum of Zoology, Senckenberg Dresden, Dresden, Germany; 3 Turtle Island, Graz, Austria; 4 landsnails.org, Prague, Czech Republic; 5 Prague Zoological Garden, Prague, Czech Republic

**Keywords:** Evolutionary genetics, Herpetology

## Abstract

Turtles demonstrate variability in sex determination and, hence, constitute an excellent model for the evolution of sex chromosomes. Notably, the sex determination of the freshwater turtles from the family Chelidae, a species-rich group with wide geographical distribution in the southern hemisphere, is still poorly explored. Here we documented the presence of an XX/XY sex determination system in seven species of the Australasian chelid genera *Chelodina*, *Emydura*, and *Elseya* by conventional (karyogram reconstruction, C-banding) and molecular cytogenetic methods (comparative genome hybridization, *in situ* hybridization with probes specific for GATA microsatellite motif, the rDNA loci, and the telomeric repeats). The sex chromosomes are microchromosomes in all examined species of the genus *Chelodina*. In contrast, the sex chromosomes are the 4^th^ largest pair of macrochromosomes in the genera *Emydura* and *Elseya*. Their X chromosomes are submetacentric, while their Y chromosomes are metacentric. The chelid Y chromosomes contain a substantial male-specific genomic region with an accumulation of the GATA microsatellite motif, and occasionally, of the rDNA loci and telomeric repeats. Despite morphological differences between sex chromosomes, we conclude that male heterogamety was likely already present in the common ancestor of *Chelodina*, *Emydura* and *Elseya* in the Mesozoic period.

## Introduction

Amniotes possess two major sex determination systems: genotypic sex determination (GSD) and environmental sex determination (ESD). In GSD, the sex of an individual is determined by its sex-specific genotype, i.e. the combination of sex chromosomes. On the contrary, in ESD, the sex of an individual is influenced by environmental conditions and there are no consistent genotypic differences between sexes. The most well studied type of ESD is the temperature-dependent sex determination (TSD), where the sex of the individual is influenced by the temperature during a sensitive period of embryonic development (the definitions follow Johnson Pokorná & Kratochvíl^1^). Three amniote lineages, the geckos (infraorder Gekkota), the dragon lizards (family Agamidae) and the turtles (order Testudines), show extensive variability of sex determination systems, and closely related species have either GSD or ESD^1–4^, making them excellent groups for exploring the evolution of sex determination.

Turtles include 361 currently recognized extant species^5–7^. Unfortunately, the sex determination system is known in only approximately 24% of all species, and sex chromosomes have been up to now reported for only 20 species^4,8–10^. Phylogenetic reconstruction of sex determination systems suggested that ESD is ancestral in turtles and sex chromosomes, and thus GSD, evolved at least five times independently. In the suborder Cryptodira, XX/XY sex chromosomes have been reported for *Siebenrockiella crassicollis* (family Geoemydidae)^4,11,12^ and for the genera *Staurotypus* (family Kinosternidae)^13^ and *Glyptemys* (family Emydidae)^14,15^. In contrast, ZZ/ZW sex chromosomes are widely shared in softshell turtles (family Trionychidae)^9,16,17^. Recently, we demonstrated that the report on ZZ/ZW sex chromosomes in *Pangshura smithii* (Geoemydidae)^18^ was based on the erroneous pairing of chromosomes in the karyogram, and that this species has either GSD with poorly differentiated sex chromosomes or ESD^19^.

In the suborder Pleurodira, GSD was previously described for a few freshwater turtles of the family Chelidae^20–22^, a group consisting of 58 currently recognized species^5^. Chelid turtles form two geographically distinct clades, one distributed in Australasia and the other in South America^23–27^. Members of the family Chelidae have generally high diploid chromosome numbers ranging from 2n = 48 to 2n = 64^10,20^. Stable sex ratios of hatchlings incubated across a range of constant temperatures suggest the presence of GSD in at least three chelid species (*Mesoclemmys gibba*, *Phrynops geoffroanus*, *Phrynops hilarii*)^8^. Cytogenetic studies reported differentiated XX/XY sex chromosomes in three additional species, namely in *Acanthochelys radiolata*, *Chelodina longicollis*, and *Emydura macquarii*^20–22,28^. In *A. radiolata*, the pair of sex chromosomes consists of a medium-sized metacentric and a small acrocentric chromosome^20^. However, McBee *et al*.^20^ examined just a single individual (male), and the authors could neither determine the X or the Y chromosome in the heteromorphic pair, nor test whether this heteromorphism is linked to sex. Therefore, we consider the report on sex chromosomes in *A. radiolata* dubious. The sex chromosomes in *Chelodina longicollis* were identified as a pair of small chromosomes with a subtelocentric X and a submetacentric Y chromosome^21^. However, based on the accumulation of the GATA microsatellite motif, Matsubara *et al*.^28^ identified the Y chromosome in the same species as another, notably smaller microchromosome. The sex chromosomes in *Emydura macquarii* form the fourth-largest pair in the karyogram, consisting of a metacentric X and a submetacentric Y chromosome^22^, with a prominent C-positive band in the telomeric region of the short (p) chromosome arm^28^. In contrast to turtles from the family Chelidae, temperature-dependent sex determination was previously reported in species from the pleurodiran families Pelomedusidae (*Pelomedusa subrufa, Pelusius castaneus*) and Podocnemididae (*Podocnemis unifilis*, *Podocnemis expansa*, and *Podocnemis erythrocephala*)^8,29–32^.

In the current study, we explored sex chromosomes and karyotypes in the side-necked turtles of the genera *Chelodina* (*C. expansa, C. novaeguineae, C. mccordi, C. reimanni, C. rugosa*), *Emydura* (*Em. macquarii krefftii*), *Elseya* (*El. novaeguineae*), and two sibling individuals of the intergeneric hybrid *Em. subglobosa* (♀) × *El. novaeguineae* (♂) by applying a combination of conventional and molecular cytogenetic methods. We reconstructed karyograms and examined the presence of differentiated sex chromosomes by C-banding, comparative genome hybridization (CGH), and fluorescence *in situ* hybridization (FISH) with probes specific for GATA motif, telomeric repeats and rDNA loci, i.e. repetitive elements which often accumulate on the sex chromosomes of reptiles^15,28^.

## Results

### Species verification

The 5′ end of the mitochondrial cytochrome c oxidase I gene (COI) and/or the mitochondrial cytochrome b gene (cytb) were successfully amplified and sequenced and whenever possible compared to sequences from type specimens recently published by Kehlmaier *et al*.^33^. All studied individuals showed distinctly less than 3% genetic p-distance from the respective type specimens of the species with which they were identified. However, *C. novaeguineae* and *C. reimanni* do not differ in their mitochondrial DNA and the validity of *C. reimanni* is doubtful^33^. Accordingly, the COI of our specimens of *C. reimanni* was identical with the type sequences of these two species and our material was identified based on morphology.

### Karyotype reconstruction and heterochromatin distribution

**Figure 1:**
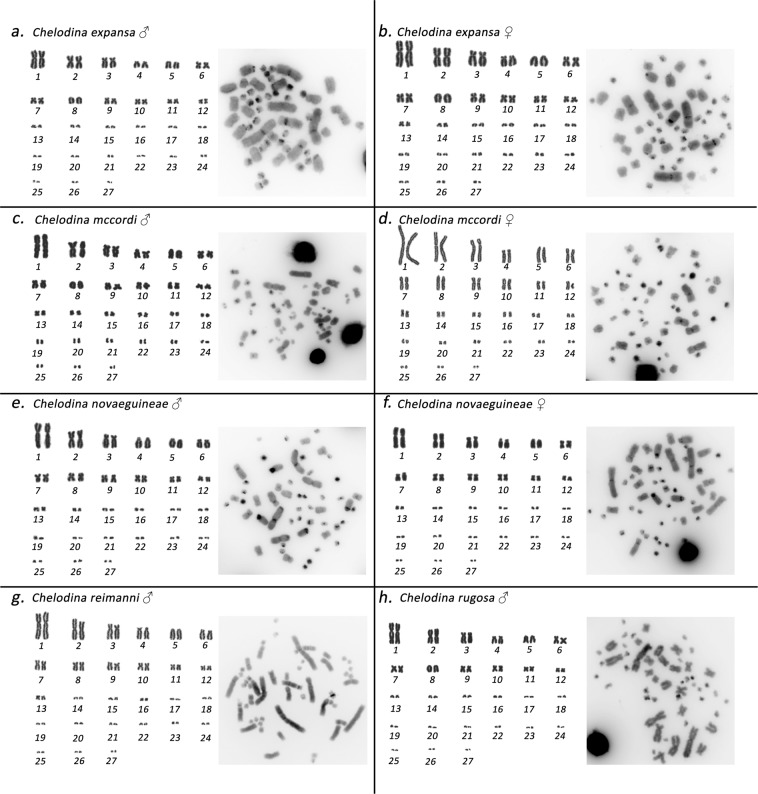
Giemsa-stained karyotype and C-banded metaphases in *Chelodina expansa* (**a,b**), *Chelodina mccordi* (**c,d**), *Chelodina novaeguineae* (**e,f**), *Chelodina reimanni* (**g**), and *Chelodina rugosa* (**h**). The pairing of microchromosomes does not indicate homology but morphological similarity.

All examined individuals of the genus *Chelodina* had similar karyotypes with 2n = 54 chromosomes consisting of 12 pairs of macrochromosomes and 15 pairs of microchromosomes. All macrochromosomes were bi-armed, with the exception of the acrocentric chromosome pairs 5 and 8 in *C. expansa, C. mccordi*, and *C. rugosa* and of chromosome pair 5 in *C. novaeguineae* and *C. reimanni* (Fig. 1). C-banding stain revealed constitutive heterochromatin in the centromeric regions of all chromosomes. In addition, heterochromatic blocks were detected in up to four pairs of microchromosomes in all species as well as in the p-arms of the submetacentric chromosomes from the 4^th^ pair in *C. novaeguineae* (Fig. 1).

**Figure 2:**
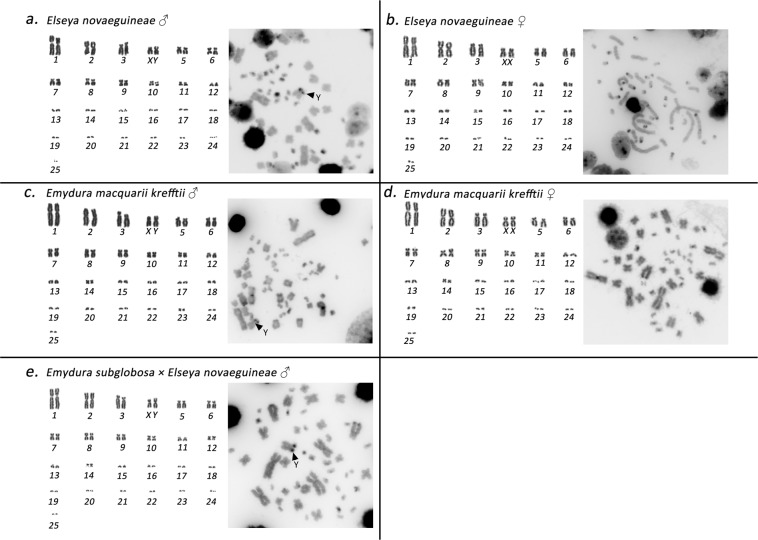
Giemsa-stained karyotype and C-banded metaphases in *Elseya novaeguineae* (**a,b**), *Emydura macquarii krefftii* (**c,d**), and the hybrid *Em. subglobosa* × *El. novaeguineae* (**e**). The pairing of microchromosomes does not indicate homology but morphological similarity.

The individuals from the genera *Emydura* and *Elseya* possessed karyotypes with 2n = 50 chromosomes consisting of 12 pairs of macrochromosomes and 13 pairs of microchromosomes. All macrochromosomes were bi-armed. The 4^th^ largest chromosome pair consisted of two submetacentric chromosomes in females, but a metacentric chromosome and a submetacentric chromosome in males (*El. novaeguineae, Em. macquarii krefftii*, and the two male hybrids *Em. subglobosa* × *El. novaeguineae*; Fig. 2). C-banding revealed constitutive heterochromatin in the centromeric regions of all chromosomes. In addition, heterochromatic blocks were observed in four pairs of microchromosomes and in the 4^th^ largest pair (Fig. 2).

### *In situ* hybridization with probes for GATA motif, telomeric repeats and rDNA loci

**Figure 3:**
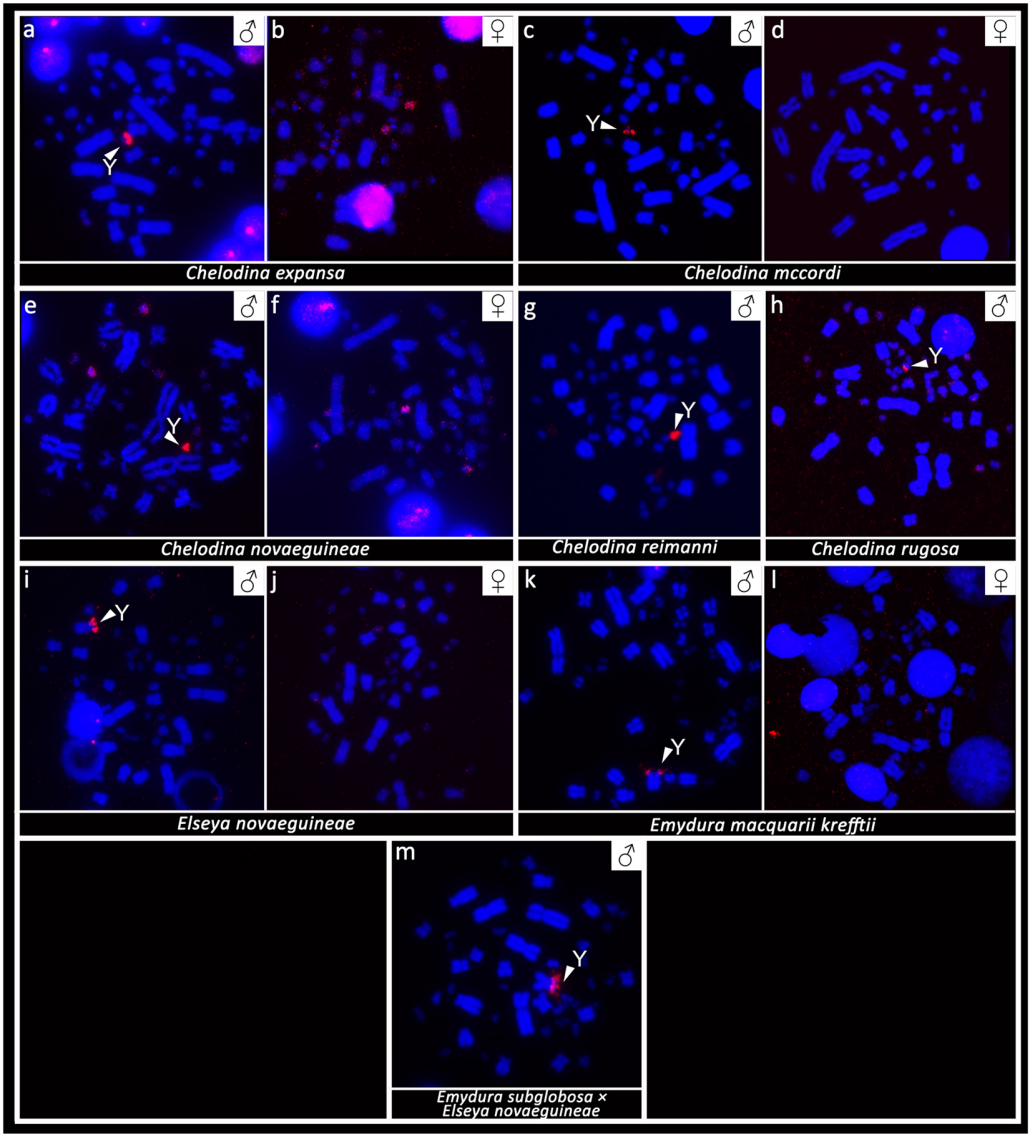
*In situ* hybridization with the probe specific for the (GATA)_8_ microsatellite motif in *Chelodina expansa* (**a,b**), *Chelodina mccordi* (**c,d**), *Chelodina novaeguineae* (**e,f**), *Chelodina reimanni* (**g**), *Chelodina rugosa* (**h**), *Elseya novaeguineae* (**i,j**), *Emydura macquarii krefftii* (**k,l**), and the hybrid *Em. subglobosa* × *El. novaeguineae* (**m**). The FITC signal of the GATA probe was pseudocolourized in red. All metaphases were counterstained with DAPI (blue). The Y chromosome is indicated with a white arrow.

FISH with probes specific for the GATA microsatellite motif revealed a strong accumulation in a single microchromosome in the males of the genus *Chelodina*. Strong accumulations of this motif were revealed in all males of the genera *Emydura* and *Elseya* in the heterochromatic region in the terminal position of the p-arm of the metacentric chromosome from the 4^th^ pair. No accumulation of the GATA motif was detected in females (Fig. 3) with the only exception of *C. expansa*, where the accumulation of the GATA microsatellite motif was identified in three microchromosomes in males but in only two microchromosomes in females.

**Figure 4:**
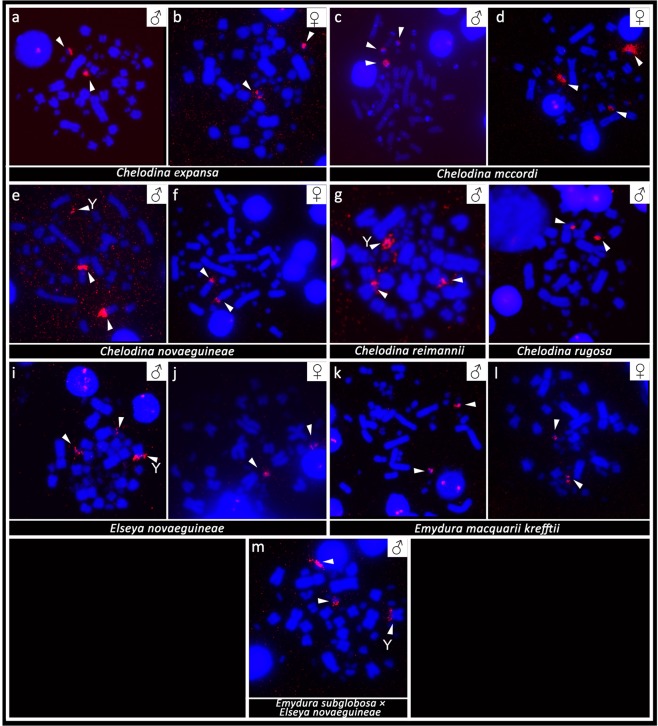
*In situ* hybridization with probe specific for the (TTAGGG)_n_ telomeric motif in *Chelodina expansa* (**a,b**), *Chelodina mccordi* (**c,d**), *Chelodina novaeguineae* (**e,f**), *Chelodina reimanni* (**g**), *Chelodina rugosa* (**h**), *Elseya novaeguineae* (**i,j**), *Emydura macquarii krefftii* (**k,l**), and the hybrid *Em. subglobosa* × *El. novaeguineae* (**m**). The FITC signal was pseudocolourized in red. All metaphases were counterstained with DAPI (blue). The Y chromosome is indicated with a white arrow.

The probe specific for the telomeric repeats revealed the expected terminal topology. In addition, strong accumulation of telomeric-like motifs was detected in microchromosomes in all studied individuals as well as in the terminal position of the p-arm of the metacentric chromosome from the 4^th^ pair in *the hybrid Em. subglobosa* × *El. novaeguineae* (Fig. 4).

**Figure 5:**
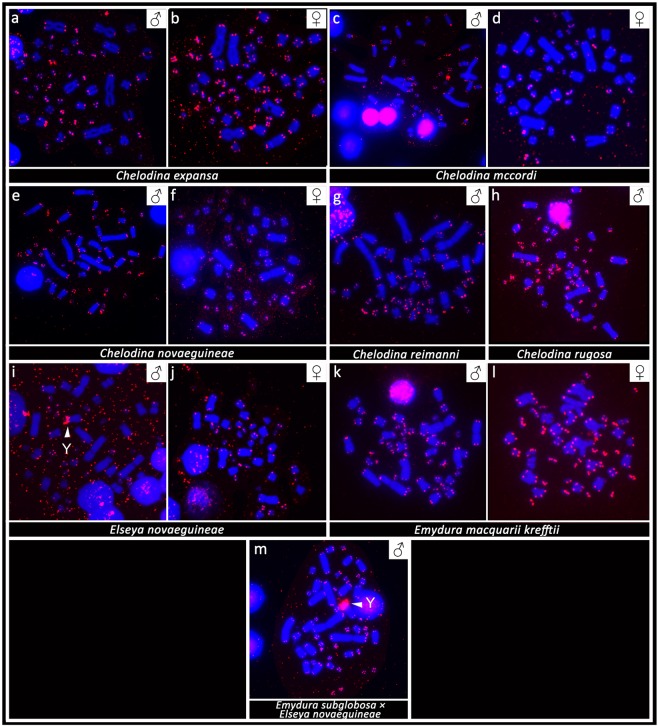
*In situ* hybridization with probe specific for the rDNA sequence in *Chelodina expansa* (**a,b**), *Chelodina mccordi* (**c,d**), *Chelodina novaeguineae* (**e,f**), *Chelodina reimanni* (**g**), *Chelodina rugosa* (**h**), *Elseya novaeguineae* (**i,j**), *Emydura macquarii krefftii* (**k,l**), and the hybrid *Em. subglobosa* *×* *El. novaeguineae* (**m**). The FITC signal was pseudocolourized in red. All metaphases were counterstained with DAPI (blue). The Y chromosome is indicated with a white arrow.

FISH with probes specific for the rDNA loci showed strong accumulation in two chromosomes in both sexes of *C. expansa* and *C. rugosa*. rDNA loci were accumulated in two chromosomes in females of *C. novaeguineae*, but in three chromosomes in males of *C. novaeguineae* and *C. reimanni*. Notably, an accumulation of rDNA loci was detected in three microchromosomes in both sexes of *C. mccordi*. rDNA loci accumulated in two microchromosomes in females of *El. novaeguineae* and *Em. macquarii krefftii*. In addition, rDNA loci accumulated also in the telomeric position of the p-arm of the metacentric chromosome from the 4^th^ pair in male turtles of *El. novaeguineae* and the *Em. subglobosa* × *El. novaeguineae* hybrids but not in the homologous chromosome of *Em. macquarii krefftii* (Fig. 5).

### Comparative genome hybridization

**Figure 6:**
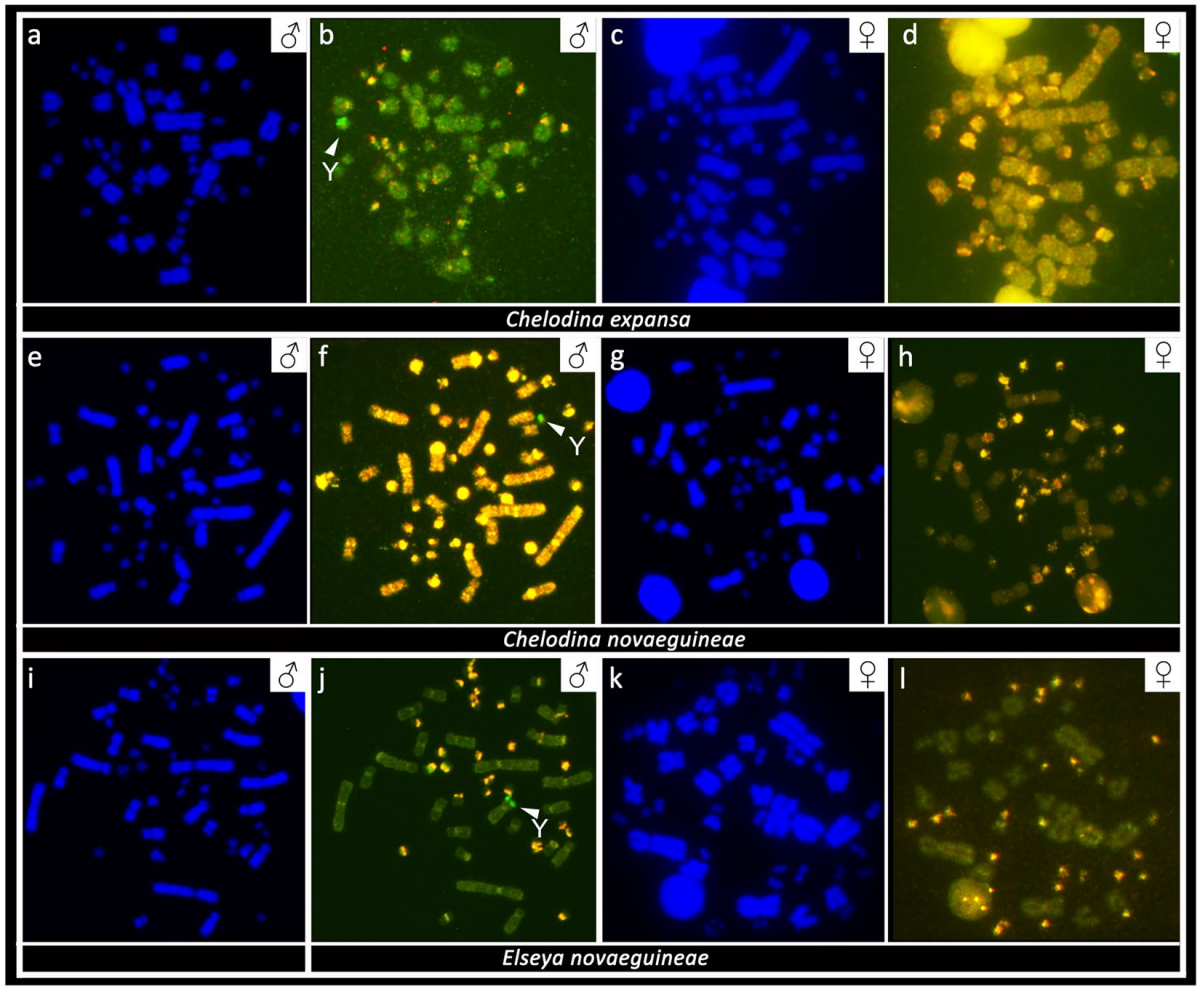
Comparative genome hybridization with FITC-labelled probe specific for male (green) and rhodamine-labelled probe specific for female (red) genomic content in *Chelodina expansa* (**a–d**), *Chelodina novaeguineae* (**e–h**), and *Elseya novaeguineae* (**i–l**). Chromosomal regions with similar genomic content between sexes are visualized in yellow. The white arrow indicates male-specific region (green), corresponding to the Y chromosome.

CGH revealed strong male-specific genomic content in a single microchromosome in metaphases from males of *C. expansa* and *C. novaeguineae*. Male-specific genomic content was detected at the terminal position of the p-arm of the metacentric chromosome from the 4^th^ pair in metaphases from males of *El. novaeguineae*. No sex-specific content was found in metaphases of females in *C. expansa*, *C. novaeguineae* and *El. novaeguineae* (Fig. 6).

## Discussion

Freshwater turtles of the family Chelidae have karyotypes with 2n = 54 chromosomes in the genus *Chelodina* and 2n = 50 chromosomes in the genera *Elseya* and *Emydura* (Figs. 1 and 2). Our cytogenetic examination confirmed previously published karyotypes for *C. expansa, C. novaeguineae, C. rugosa* and *Em. m. krefftii*, with respect to chromosome numbers and morphology^10^, while karyotypes for *C. mccordi*, *C. reimanni*, and *El. novaeguineae* are presented here for the first time (Figs. 1 and 2). Within the genus *Chelodina* there was an evolutionary change in the shape of the chromosome pair 8, which is metacentric in *C. novaeguineae* and *C. reimanni*, but acrocentric in *C. expansa, C. mccordi* and *C. rugosa*. The metacentric shape in *C. novaeguineae* and *C. reimanni* can be a synapomorphy of these closely related or synonymous species^33^. The transitions between acrocentric and metacentric shape in a chromosome pair together with the conservation in chromosome numbers are often caused by intrachromosomal rearrangements in reptiles^34–37^. This hypothesis should be tested in the genus *Chelodina* by comparative cytogenetics in future, using whole chromosome painting or comparative BAC-FISH.

*In situ* hybridization with probes specific for repetitive elements that often accumulate on vertebrate sex chromosomes revealed an extensive accumulation of the GATA microsatellite motif in odd numbers of chromosomes in the metaphases of all male chelids. The relevant chromosome corresponds to a dot-like microchromosome in the genus *Chelodina* but to a single chromosome of the 4^th^ pair of the complement in the genera *Emydura* and *Elseya* (Fig. 3). In addition, rDNA loci are amplified in odd numbers of chromosomes in males of *C. novaeguineae*, *C. reimanni*, *El. novaeguineae* and in the *Em. subglobosa* × *El. novaeguineae* hybrids (Fig. 4). Telomeric repeats seem to accumulate in a single chromosome of the 4^th^ pair of the complement in *El. novaeguineae* and in the *Em. subglobosa* × *El. novaeguineae* hybrids (Fig. 5). We suggest that the chromosome with the amplification of the GATA microsatellite motif and in some species also of the rDNA loci and telomeric-like repeats in males is the Y chromosome. This conclusion is further supported by the results of CGH performed here for three species (*C. expansa*, *C. novaeguineae*, and *El. novaeguineae*) visualizing a male-specific genomic content within these chromosomes (Fig. 6). A metacentric Y chromosome was previously described for *Em. m. macquarii* by Martinez *et al*.^22^ with similar morphology as in *El. novaeguineae, Em. macquarii krefftii* and the two male *Em. subglobosa* × *El. novaeguineae* hybrids. In contrast to *El. novaeguineae* and the two male *Em. subglobosa* × *El. novaeguineae* hybrids, the accumulation of GATA microsatellite repeats was not detected here in the metacentric Y chromosome of *Em. macquarii krefftii* and previously also in *Em. m. macquarii*^28^. We assume that the GATA motif does not exist or accumulates in very low copy numbers in the Y chromosome of *Em. macquarii krefftii* and *Em. m. macquarii*, below the detection threshold of molecular cytogenetic methods. This situation likely reflects the extensive evolutionary dynamics of the heterochromatic content of degenerated sex chromosomes in sauropsids^28,38,39^.

As identified with our cytogenetic methods (i.e. karyotype reconstruction, C-banding, and FISH), the X chromosome is the submetacentric chromosome in the 4^th^ largest pair of the complement in *Elseya* and *Emydura* (Fig. 2). We were not able to visualize the X chromosome in the genus *Chelodina* by our cytogenetic methods, yet, the chromosome pairing in karyograms suggests that it should be a microchromosome (Fig. 1). Ezaz *et al*.^21^ concluded that sex chromosomes in *C. longicollis* correspond to a pair of small-sized chromosomes with a prominent heterochromatic block. However, we assume that the sex chromosomes of *C. longicollis* were misidentified in the study of Ezaz *et al*.^21^. Our results agree with Matsubara *et al*.^28^ who showed the Y chromosome in *Chelodina* is a different, tiny microchromosome with a prominent amplification of microsatellite repeats.

**Figure 7:**
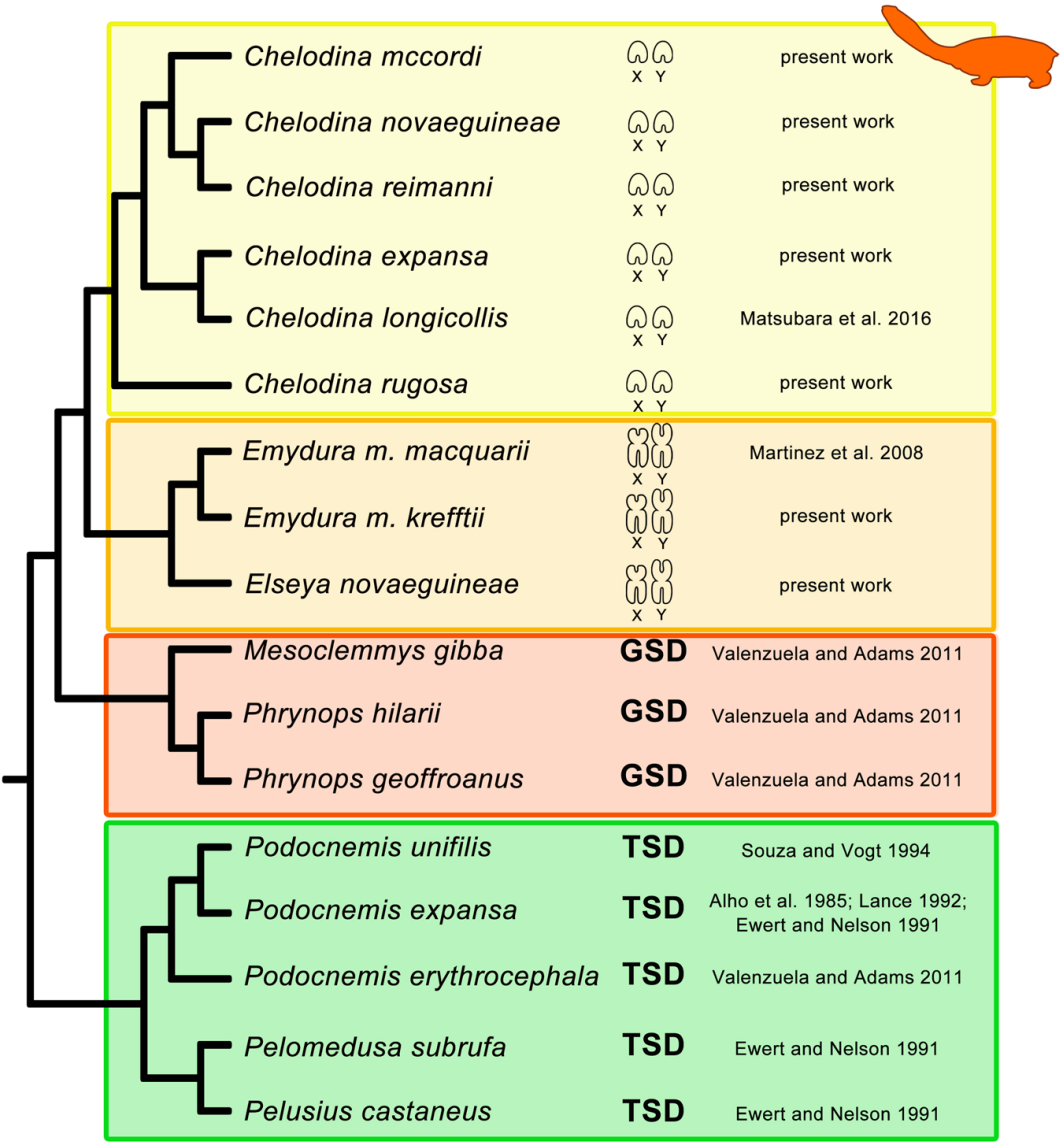
Overview of current knowledge on sex determination in side-necked turtles. Phylogeny follows Valenzuela & Adams^8^ and Kehlmaier *et al*.^33^. Information on sex determination systems was compiled from this and previously published studies^8,21,22,28–32^. Sex chromosomes are microchromosomes in turtles from the genus *Chelodina*, but macrochromosomes in turtles from the genera *Elseya* and *Emydura*. GSD: genotypic sex determination, TSD: temperature dependent sex determination.

All Australasian chelid species studied to date possess an XX/XY sex determination system (this study^21,22,28^). Homology between XX/XY sex chromosomes with dissimilar morphology in representatives from the genus *Chelodina* when compared with *Elseya* and *Emydura* might be supported by accumulation of the same repetitive motifs (GATA microsatellite, rDNA, telomeric-like sequences) in at least some members of these two clades (Figs. 3–5), but the accumulation of the same repetitive motifs in heterochromatic regions is generally a poor indicator of sex chromosome homology^28,39^. Matsubara *et al*.^28^ suggested that the sex chromosomes in the ancestor of Australasian chelids were a pair of microchromosomes, similar to the recent *Chelodina*, and a rearrangement occurred in the common ancestor of *Elseya* and *Emydura*. According to Matsubara *et al*.^28^, the ancestral sex chromosomes either (i) fused with a medium-sized pair of autosomes or (ii) a part of the ancestral sex chromosomes, including the sex determining region and a surrounding repetitive content, was translocated to a medium-sized autosome. However, the clades of the genera *Chelodina* and *Elseya/Emydura* show a sister group relationship (Fig. 7). Therefore, we assume that another scenario for sex chromosome homology is equally parsimonious, i.e. that the ancestral sex chromosomes were of the *Elseya*/*Emydura* type and a chromosomal rearrangement in the ancestor of the genus *Chelodina* transferred the sex-determining locus to a microchromosome.

If sex determination is homologous between the two chelid clades, the XX/XY chromosomes in this group could date back to their last common ancestor living *c*. 50–120 million years ago^27,40,41^. In order to scrutinize the possible homology of sex chromosomes across Australasian chelids in future, it will be crucial to identify the gene content of their sex chromosomes using genomic methods, as recently applied in other reptilian lineages^9,42–46^.

## Material and Methods

### Studied material

We collected blood samples to establish cell cultures for chromosome preparations and for DNA isolation from side-necked turtles of the genera *Chelodina* (*C. expansa, C. mccordi, C. novaeguineae, C. reimanni, C. rugosa*), *Emydura* (*Em. macquarii krefftii*) and *Elseya* (*El. novaeguineae*), and two sibling hybrids *Em. subglobosa* (♀) × *El. novaeguineae* (♂). All turtles are either captive-bred or legally imported from the wild, and kept at Plzeň Zoo (Czech Republic), the Zoo Prague (Czech Republic), Turtle Island (Austria), or the Museum of Zoology, Senckenberg Dresden (Germany). A detailed list of specimens is provided in Table 1. Blood samples were collected by veterinarians primarily for diagnostic purposes, which is not considered as an experiment on animals according to Czech legislation (No. 46/1992). The owners of the turtles approved the use of blood samples for the current study. All methods were carried out in accordance with relevant guidelines and regulations, by researchers accredited for animal experimental design by the Ministry of Agriculture of the Czech Republic (Lukáš Kratochvíl: accreditation CZ02535; Michail Rovatsos: accreditation CZ03540), and with the approval of the Ethical Committee of Faculty of Science, Charles University.

**Table 1:** List of individuals analyzed in this study. Diploid chromosome number (2n) and sex are indicated.

Species	2n	♂	♀
*Chelodina expansa*	54	1	2
*Chelodina mccordi*	54	2	2
*Chelodina novaeguineae*	54	3	2
*Chelodina reimanni*	54	2	—
*Chelodina rugosa*	54	2	—
*Elseya novaeguineae*	50	1	1
*Emydura macquarii krefftii*	50	1	2
*Em. subglobosa* (♀) × *El. novaeguineae* (♂)	50	2	—

### DNA isolation, chromosome preparation and staining

Genomic DNA was extracted from blood samples using a DNeasy Blood and Tissue Kit (Qiagen). Chromosome suspensions for cytogenetic analyses were obtained from whole-blood lymphocyte cultures following the protocol described in Mazzoleni *et al*.^19^. Chromosome spreads were stained with Giemsa for karyotype reconstruction (Figs. 1 and 2). The distribution of constitutive heterochromatin was detected by C-banding^47^, with slight modifications as described in Mazzoleni *et al*.^19^.

### Species verification

Species identification is challenging in the genus *Chelodina*, as taxonomy is complicated and not fully resolved yet (for review see Kehlmaier *et al*.^33^). Therefore, we characterized our material and verified its taxonomy by sequencing the standard “DNA barcoding” region from the mitochondrial cytochrome c oxidase subunit I gene (COI) and/or the mitochondrial cytochrome b gene (cytb). This data is intended to genetically identify our cytogenetic material in future, regardless of potential taxonomic changes. The COI fragment was amplified by PCR using either the reptile-specific primers RepCOI-F and RepCOI-R^48^ or the universal primers LCO1490 and HCO2198^49^. The cytb gene was amplified by PCR using the primers H16064 and L14919^50^. For both genes, we prepared the PCR reaction and cycling conditions according to Koubová *et al*.^51^. The PCR products were sequenced bi-directionally by Macrogen (Korea), and the obtained haplotype sequences were deposited in GenBank under the accession numbers MN757883-MN757886. The COI and cytb sequences were aligned using CLUSTALW^52^, as implemented in BioEdit v5.0.9^53^, and subsequently analyzed in DnaSP v5.10.1^54^. All sequences were compared with those from Kehlmaier *et al*.^33^, derived from type specimens, and Le *et al*.^55^. Genetic distances among haplotypes were calculated in MEGA v7^56^.

### Fluorescence *in situ* hybridization

The distributions of the GATA microsatellite motif was examined, as well as of the TTAGGG telomeric repeat and the rDNA loci, using FISH. The (GATA)_8_ probe was synthesized and labeled with biotin (Macrogen, Korea). The telomeric probe was synthesized and labeled with biotin by PCR according to a previously published protocol^57^. The probe for the rDNA loci was prepared from a plasmid (pDm r.a51#1) with an 11.5-kb insertion, encoding the 18S and 28S rRNA units of *Drosophila melanogaster*^58^; for the labeling protocol see Rovatsos *et al*.^9^. Hybridization conditions, post-hybridization washes, signal amplification and detection are explained in detail in Rovatsos *et al*.^59^.

### Comparative genome hybridization

To detect sex-specific regions of the genome, CGH was performed using metaphase chromosomes of both male and female individuals of *C. expansa, C. novaeguineae*, and *El. novaeguineae*. The detailed protocol for probe and hybridization experiments is presented in Rovatsos *et al*.^59^.

### Microscopy and image analysis

Giemsa-stained metaphase chromosomes were studied under a Carl Zeiss AxioImager.Z2 microscope, equipped with Metafer Scanning Platform (Metasystems) and a MetaSystems CoolCube digital camera. Images were processed for karyotype reconstruction with Ikaros karyotyping software (Metasystems). For C-banding, FISH and CGH methods, images from at least 20 metaphase chromosomes were analyzed using a Provis AX70 (Olympus) fluorescence microscope, equipped with a DP30BW digital camera (Olympus). All images were acquired in black and white, and later superimposed with colours in DP Manager imaging software (Olympus).
