# Author Correction: Sex is determined by XX/XY sex chromosomes in Australasian side-necked turtles (Testudines: Chelidae)

**DOI:** 10.1038/s41598-020-63116-2

**Published:** 2020-04-03

**Authors:** Sofa Mazzoleni, Barbora Augstenová, Lorenzo Clemente, Markus Auer, Uwe Fritz, Peter Praschag, Tomáš Protiva, Petr Velenský, Lukáš Kratochvíl, Michail Rovatsos

**Affiliations:** 1 Department of Ecology, , Faculty of Science, Charles University, Viničná 7, Prague, Czech Republic; 2 Museum of Zoology, Senckenberg Dresden, Dresden, Germany; 3 Turtle Island, Graz, Austria; 4 landsnails.org, Prague, Czech Republic; 5 Prague Zoological Garden, Prague, Czech Republic

Correction to: *Scientific Reports* 10.1038/s41598-020-61116-w, published online 09 March 2020

This Article contains an error in the order of the Figures. Figures 4 and 5 were published as Figures 5 and 4 respectively. The correct Figures 4 and 5 appear below as Figs. 1 and 2. The Figure legends are correct.

**Figure 1:**
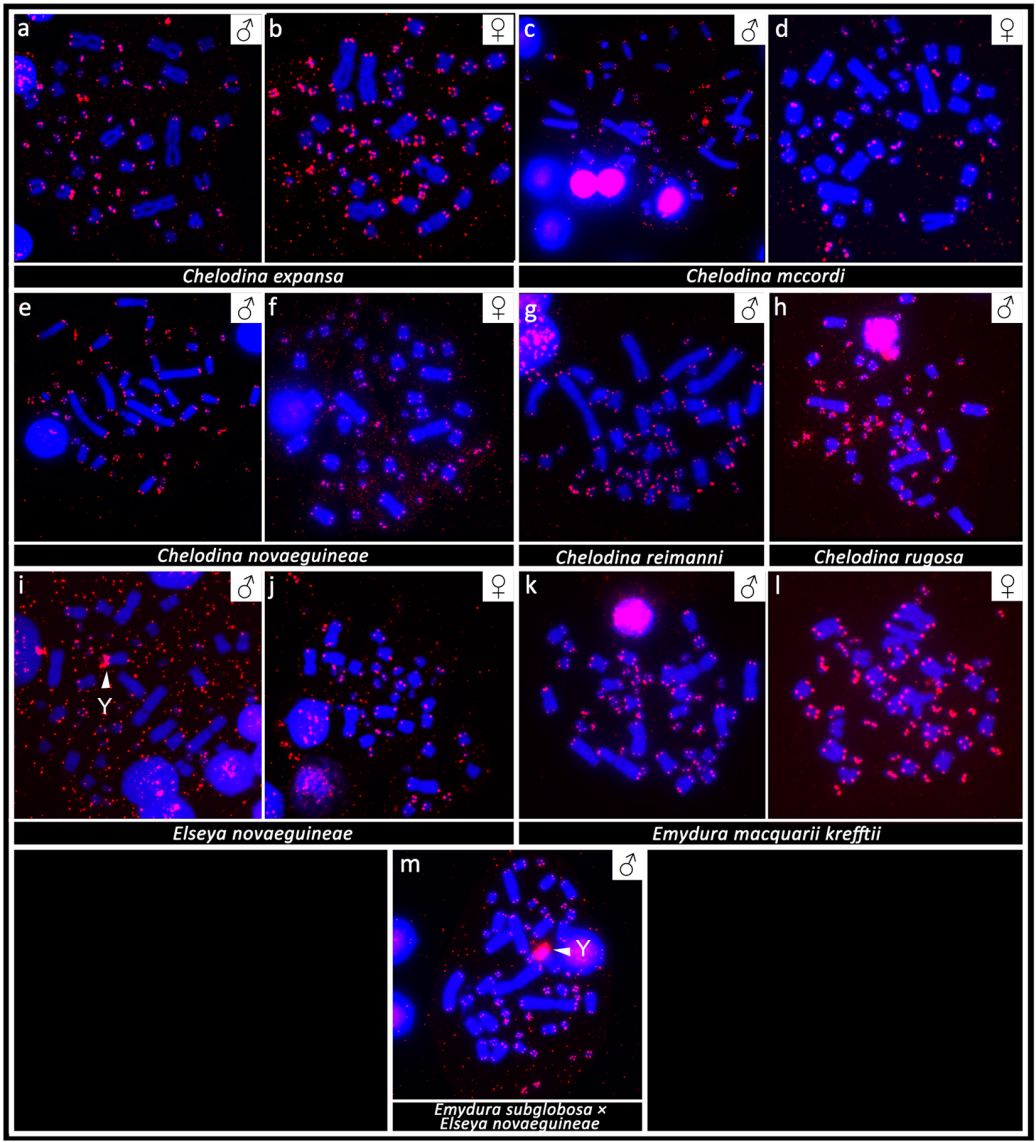
*In situ* hybridization with probe specific for the (TTAGGG)n telomeric motif in Chelodina expansa (**a**,b), Chelodina mccordi (**c,d**), Chelodina novaeguineae (**e,f**), Chelodina reimanni (**g**), Chelodina rugosa (**h**), Elseya novaeguineae (**i,j**), Emydura macquarii krefftii (**k,l**), and the hybrid Em. subglobosa × El. novaeguineae (**m**). The FITC signal was pseudocolourized in red. All metaphases were counterstained with DAPI (blue). The Y chromosome is indicated with a white arrow.

**Figure 2:**
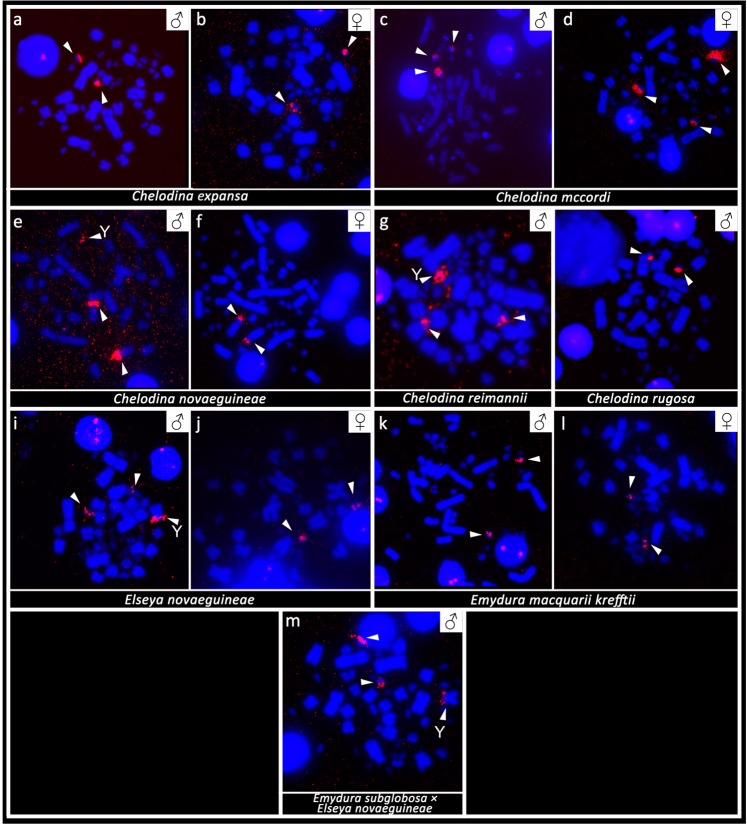
*In situ* hybridization with probe specific for the rDNA sequence in Chelodina expansa (**a,b**), Chelodina mccordi (**c,d**), Chelodina novaeguineae (**e,f**), Chelodina reimanni (**g**), Chelodina rugosa (**h**), Elseya novaeguineae **(i,j**), Emydura macquarii krefftii (**k,l**), and the hybrid Em. subglobosa × El. novaeguineae (**m**). The FITC signal was pseudocolourized in red. All metaphases were counterstained with DAPI (blue). The Y chromosome is indicated with a white arrow.

